# Our children deserve cleaner air

**DOI:** 10.1016/j.eclinm.2025.103679

**Published:** 2025-11-20

**Authors:** eClinicalMedicine

A new study published in *eClinicalMedicine* on Nov 1, 2025, highlights the devastating impact of air pollution, specifically nitrogen dioxide (NO_2_), on paediatric health. Using data collected by the Institute for Health Metrics and Evaluation as part of the Global Burden of Disease (GBD) study 2023, Katrin Burkart and colleagues derived the asthma burden attributable to NO_2_ exposure in children and young people (aged <20 years) in 1990–2023 across 204 countries and territories. They estimate that, whilst NO_2_-attributable asthma is predominantly an urban phenomenon and there are substantial variations among GBD super-regions, NO_2_ contributed 233 000 years lived with disability from paediatric asthma in 2023. These findings come against a backdrop of data from UNICEF's State of Global Air 2024 report, in which air pollution remains the second leading risk factor for death among children under 5 years; demonstrating that far too many children globally breathe air that violates WHO Global Air Quality Guidelines. With such stark consequences for child health, why are we not doing more to curb air pollution?

Air pollution refers to contamination of both ambient and household air by harmful pollutants, such as nitrogen oxides (NO_x_), polycyclic aromatic hydrocarbons, carbon monoxide (CO), sulphur dioxide (SO_2_), ground-level ozone (O_3_) and, most commonly, fine particulate matter (PM_2.5_) and its primary component of black carbon. Ambient pollutants are mainly derived from fossil fuel combustion, industrial processes, and natural processes (eg, wildfires and dust storms), with agricultural practices (India and Pakistan) and waste incineration (Bangladesh) adding to the burden in some of the most polluted regions. India, for example, has recently reported real-time highs of 500 (including on the day of writing: Nov 7, 2025), which is the maximum value India's Air Quality Index (AQI) captures; daily averages for Delhi in 2024 and 2025 exceeded healthy limits of 0–50 AQI without exception. Household air pollution describes similar hazardous particles from incomplete burning of solid fuels (eg, coal for cooking or heating). Household air pollution accounts for almost 11% of global mortality in children under 5 years, and disproportionately affects low-income and middle-income countries.

Children are uniquely vulnerable to the damaging effects of air pollution through a combination of behavioural, environmental, and physiological factors. For example, children breathe faster and intake more air than adults, resulting in greater inhalation of pollutants, and their developing organs are more vulnerable to inflammation and damage. Exposure has been linked to adverse birth outcomes, infant morbidity (including poor lung function, allergic diseases, and neurodevelopmental disorders), and infant mortality, plus IQ loss, paediatric cancers, elevated risks for chronic diseases in adulthood, and poor sleep quality. The damaging effects of air pollution upon children begin during pregnancy, with black carbon particles found in the cord blood of fetuses for the first time in 2022.

In recognising NO_2_ pollution as a key environmental risk for paediatric health, Burkart and colleagues call for targeted policy interventions and highlight that reducing NO_2_ exposure to theoretical minimums could reduce global asthma risk by 4.67%. Similarly, the World Bank calls for targeted policy action and global unity, estimating that policies that integrate decarbonisation and air quality management could, by 2040, half the number of people exposed to dangerous concentrations of PM_2.5_. Highlighting the positive impact that improving air quality could have on health.

Consistent with these calls for stronger policies, the European Public Health Alliance recommend that the EU formulate a harmonised EU framework for indoor air quality to protect the most vulnerable in society, including children, and called for recent EU directives on indoor environment quality and ambient air quality to be transposed into national law. The EU's ambient air quality directive is an ambitious roadmap towards the EU Zero Pollution Action Plan by 2050, aiming for air pollution reductions to non-hazardous concentrations. New projections to 2050 indicate that this ambition is necessary. Even with optimistic emission reductions, only 65% of the EU countries would comply with the PM_2.5_ target value of 5 μg m^−3^ (WHO Global Air Quality Guidelines). No countries were projected to comply with O_3_ limits of 60 μg m^−3^.

But there is hope. Positive examples of policy change can be found in Mexico, which has halved concentrations of most pollutants (1992–2010) through bolstered public transportation and vehicle emission-cutting policies, and in Rwanda and Kenya through sustainably financed clean cooking and transport solutions. The global disease burden for household air pollution has decreased due to reductions in exposure across China and south Asia, resulting in a 36% reduction in attributable deaths. Similarly, China's Action Plan showed success in rapidly reducing PM_2.5_ in 2014–19; though more recent trends (2021–24) show stagnation and O_3_ has increased, showing that renewed efforts are needed.

With a new report (*Clean air, healthy children*) from WHO and UNICEF on air pollution and children's health expected in early 2026, it is hoped that momentum will build towards true multisectoral action. However, in a time when the US Environmental Protection Agency is facing unprecedented budget and workforce cuts, including two-thirds of research staff that would otherwise be informing regulations around air quality, it is important to acknowledge that it is the same pollutants causing atmospheric warming that are threatening our (and our children's) health via contaminated air. Co-ordinated action is needed by global policy makers to integrate climate and air quality policies and drastically reduce our reliance on coal, oil, and gas. This will cut emissions and reap cleaner air to the benefit of both our children's health and the climate. As a previous report (2018) from WHO on this topic put it: children depend entirely on us—adults—to protect them from the threat of unsafe air. It is about time we did.

